# Beta-Catenin Accelerates Human Papilloma Virus Type-16 Mediated Cervical Carcinogenesis in Transgenic Mice

**DOI:** 10.1371/journal.pone.0027243

**Published:** 2011-11-07

**Authors:** Gülay Bulut, Shannon Fallen, Elspeth M. Beauchamp, Lauren E. Drebing, Junfeng Sun, Deborah L. Berry, Bhaskar Kallakury, Christopher P. Crum, Jeffrey A. Toretsky, Richard Schlegel, Aykut Üren

**Affiliations:** 1 Lombardi Comprehensive Cancer Center, Georgetown University Medical Center, Washington, D.C., United States of America; 2 Critical Care Medicine Department, Clinical Center, National Institutes of Health, Bethesda, Maryland, United States of America; 3 Histopathology Tissue Shared Resources, Georgetown University Medical Center, Washington, D.C., United States of America; 4 Department of Pathology, Georgetown University Medical Center, Washington, D.C., United States of America; 5 Department of Pathology, Harvard University, Boston, Massachusetts, United States of America; Howard University, United States of America

## Abstract

Human papilloma virus (HPV) is the principal etiological agent of cervical cancer in women, and its DNA is present in virtually all of these tumors. However, exposure to the high-risk HPV types alone is insufficient for tumor development. Identifying specific collaborating factors that will lead to cervical cancer remains an unanswered question, especially because millions of women are exposed to HPV. Our earlier work using an *in vitro* model indicated that activation of the canonical Wnt pathway in HPV-positive epithelial cells was sufficient to induce anchorage independent growth. We therefore hypothesized that constitutive activation of this pathway might function as the “second hit.” To address this possibility, we developed two double-transgenic (DT) mouse models, K14-E7/ΔN87βcat and K14-HPV16/ΔN87βcat that express either the proteins encoded by the E7 oncogene or the HPV16 early region along with constitutively active β-catenin, which was expressed by linking it to the keratin-14 (K14) promoter. We initiated tumor formation by treating all groups with estrogen for six months. Invasive cervical cancer was observed in 11% of the K14-ΔN87βcat mice, expressing activated β-catenin and in 50% of the animals expressing the HPV16 E7 oncogene. In double-transgenic mice, coexpression of β-catenin and HPV16 E7 induced invasive cervical cancer at about 7 months in 94% of the cases. We did not observe cervical cancer in any group unless the mice were treated with estrogen. In the second model, K14-HPV16 mice suffered cervical dysplasias, but this phenotype was not augmented in HPV16/ΔN87βcat mice. In summary, the phenotypes of the K14-E7/ΔN87βcat mice support the hypothesis that activation of the Wnt/β-catenin pathway in HPV-associated premalignant lesions plays a functional role in accelerating cervical carcinogenesis.

## Introduction

Cervical cancer is the second-leading cause of cancer deaths in women worldwide, and results in approximately 250,000 deaths each year from this HPV-related disease [1]. Nearly all cervical cancers are initiated by a subset of high-risk HPVs, predominantly HPV16 and HPV18 [2]. However, cervical cancers develop only in a small fraction of these women, typically many years following initial exposure. Therefore, HPV appears to be required, but insufficient for developing cervical cancer. Although recent advances in HPV prevention have been made with the introduction of HPV vaccines, they will only protect 30% of those subsequently infected with high risk HPV subtypes, and will most likely have little or no effect on patients already infected by HPV [1].

Several transgenic mouse models have been developed to investigate the biology of HPV-mediated tumorigenesis [3], [4], [5]. In one clinically relevant mouse model, the keratin-14 (K14) promoter drives the expression of the HPV E6/E7 oncogenes [6] specifically in stratified squamous epithelium, including that of the skin and cervix. These K14-HPV16 mice develop cervical pathologies only when they are treated with estrogen [7]. The phenotype appears, at 5 months of age, as increased squamous hyperplasia, epithelial papillomatosis, dysplasia, and squamous metaplasia. Microscopic invasive tumors are detected in a small fraction of the animals [8]. Cervical tumor cells in this transgenic mouse model exhibit similar biomarker expression patterns to those of humans [9]. In the second mouse model, the same K14 promoter drives HPV16-E7 oncogene expression [4], [9], which results in development of high-grade cervical dysplasia and invasive cervical malignancies in 80% of the animals [10]. It would be reasonable to conclude, therefore, that expression of the HPV16-E7 oncogene in cervical epithelial cells is sufficient to initiate oncogenesis.

Members of the Wnt family of secreted growth factors play important roles during embryogenesis by regulating proliferation, migration, tissue polarity, and organogenesis, and contribute to the development of the genitourinary system. In the canonical Wnt pathway, β-catenin acts as the central component [11]. The binding of Wnt to its receptor (frizzled) and co-receptor (LRP5/6) induces accumulation of β-catenin in the cytoplasm and nucleus. Nuclear β-catenin then binds to members of the T-cell factor (TCF) transcription factor family, leading to transcription of Wnt target genes [12]. Dysregulation of the expression of Wnt pathway components has been implicated in the development of numerous malignancies, including colon cancer, melanoma, hepatocellular carcinoma, endometrial carcinoma, ovarian carcinoma, and prostate cancer [13].

We have investigated the role of Wnt signaling in the transformation of HPV-positive human keratinocytes [14]. In this *in vitro* model, HPV immortalizes primary human keratinocytes but cannot induce malignant transformation. Therefore, the model mimics the effect of HPV on the human cervical epithelium. Activation of the canonical Wnt pathway at multiple levels (plasma membrane, cytoplasm or nucleus) specifically supports transformation of HPV-infected primary human keratinocytes. Furthermore, we and others, have detected cytoplasmic and nuclear expression of β-catenin, a hallmark of the activated Wnt pathway, in archived human cervical carcinoma samples [14], [15], [16]. We hypothesized, therefore, that activation of canonical Wnt signaling is one of the potential mechanisms facilitating cervical cancer progression in HPV-infected cells. To test this hypothesis, we generated two double-transgenic mouse models to determine whether β-catenin expression could induce tumors.

## Results

### Generation of a transgenic mouse model to study the role of the Wnt signaling in HPV pathogenesis

We used K14-E7 and K14-ΔN87βcat transgenic animals, in which the K14 promoter specifically targets expression to the cervix, to generate K14-E7/ΔN87*β*cat double-transgenic mice. K14-E7 mice express the E7 oncoprotein encoded by the high-risk HPV type 16 genome [4]. K14-ΔN87βcat mice express truncated human ΔN87βcat mRNA, which directs the synthesis of an amino-terminal truncated β-catenin molecule that lacks the four phosphorylation sites required for its degradation [17]. This mutation results in expression of constitutively active β-catenin. Under our conditions, the K14-E7 and K14-ΔN87βcat mice exhibited phenotypes similar to those reported by others [4], [17]. Thus, K14-ΔN87βcat mice develop benign skin tumors but do not exhibit histopathologic characteristics of cervical cancer. All transgenic animals were maintained as heterozygotes. Double-transgenic mice were generated by crossing K14-ΔN87βcat mice with K14-E7 mice. Crossing heterozygote K14-ΔN87βcat with heterozygote K14-E7 mice produced offspring at the expected Mendelian ratio. Only F1 generation female animals were used in the study. Tumor formation was induced by implanting estrogen pellets (0.05 mg/60 days) starting at one month of age. Each animal received 3 pellets (once every 60 days).

### K14-E7/ΔN87βcat double transgenics exhibited a more severe epidermal phenotype

**Figure 1:**
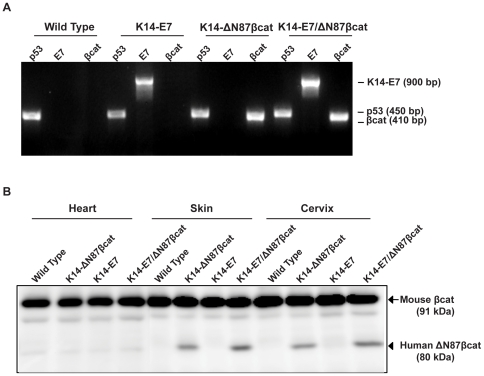
Transgenic genotypes and β-catenin expression. **A**) PCR genotyping K14-E7/ΔN87βcat mice. Genomic DNA from tail clips was used as template for primers specific for K14-E7 and K14-ΔN87βcat transgenes. DNAs amplified with *p53*-specific primers served as positive controls. Samples were electrophoresed through a 2% agarose gel and then imaged. **B**) Western blot analysis of truncated human β-catenin in K14-E7/ΔN87βcat mice. Mouse β-catenin (arrow) and human ΔN87βcat protein (arrowhead) expression in heart, skin, and cervix tissues of animals was analyzed by immunoblotting.

**Figure 2:**
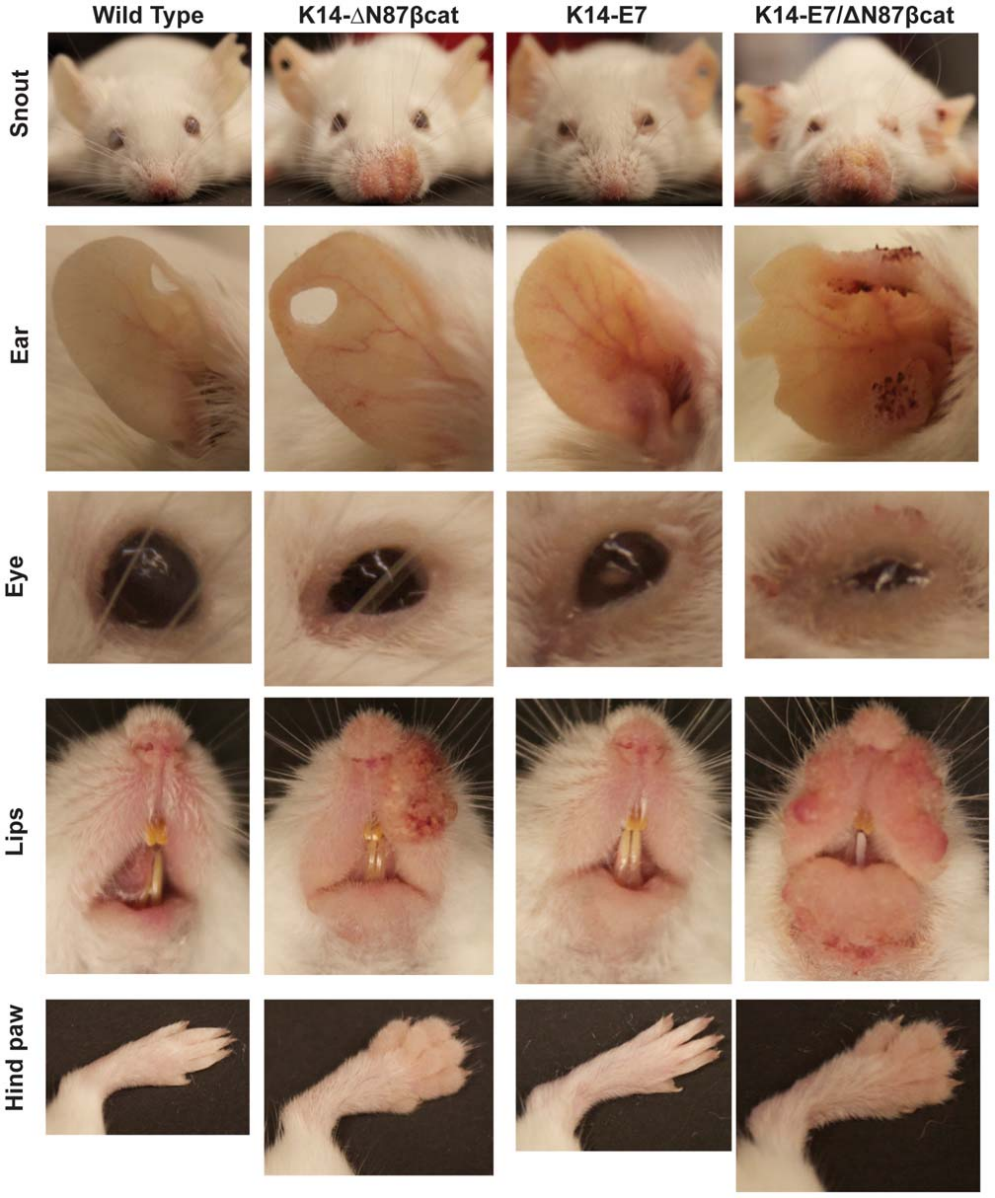
Phenotypic features of K14-E7/ΔN87βcat mice. Photographs of snout, right ear, eye, lips and hind paw are given. K14-E7/ΔN87βcat double transgenic animals displayed certain phenotypic features characteristic of both K14-E7 and K14-ΔN87βcat transgenes. Due to the combined effect of both transgenes, they had a more severe skin phenotype around the snout, ears, eyelids and lips. They had grossly enlarged paws similar to those of K14-ΔN87βcat mice.

**Figure 3:**
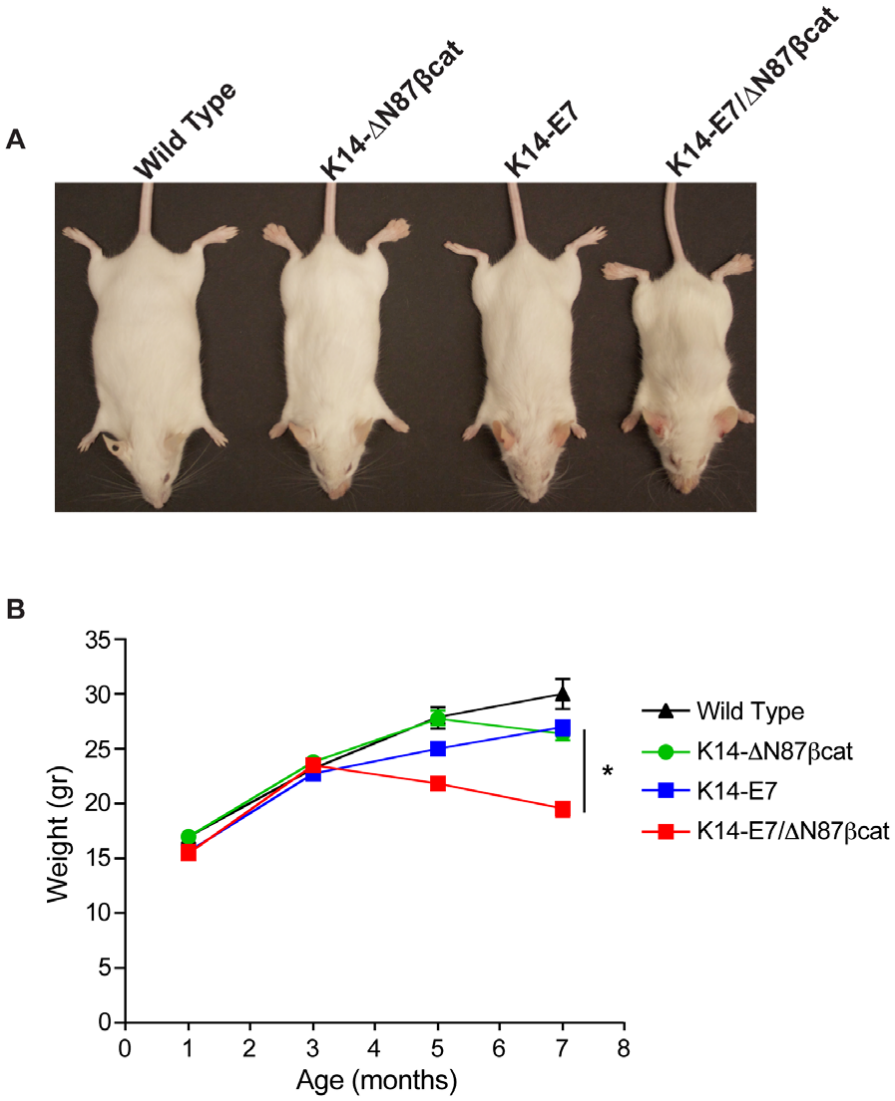
Body weights of K14-E7/ΔN87βcat mice. **A**) Whole body images of the four genotypes are given. K14-E7/ΔN87βcat animals had the lowest body weight. **B**) Lifetime weight distributions of animals representing the four genotypes are shown. The differences between the K14-E7/ΔN87βcat animals and other three genotypes were highly significant (p<0.0001). Independent *t*-test was used to determine statistical significance.

Animals were analyzed for cervical cancer development in four experimental groups: wild type, K14-ΔN87βcat, K14-E7, and K14-E7/ΔN87βcat-double transgenic animals. The genotypes of each group were confirmed by PCR analysis of genomic DNA (Figure 1A). Expression of ΔN87βcat protein in target tissues (skin and cervix) was confirmed by immunoblotting using an anti-β-catenin antibody (Figure 1B). Heart tissue, which expresses keratin 14 at very low levels, was used as a negative control. All organs analyzed from the four groups showed expression of mouse β-catenin protein (91 kDa). In contrast, expression of human ΔN87βcat was detected only in the skin and cervix of the K14-ΔN87βcat and double-transgenic animals (80 kDa) (Figure 1B). K14-E7/ΔN87βcat-double transgenic animals were viable and displayed certain phenotypic features common to K14-E7 and K14-ΔN87βcat transgenes (Figure 2) [4], [17]. Thus, K14-E7/ΔN87βcat-double transgenic exhibited a distinctive phenotype [4] characterized by wrinkled skin on their torsos, legs, and jowls, which was apparent early in postnatal life. They developed small bilateral cataracts in the eyes that were visible at weaning (Figure 2). As they aged, they exhibited thickened, less translucent ears, thick ridges around the nose and the ears, and a paucity of fur around the snout and eyes. Scaliness was apparent on their tail, ears, and feet. Their coats appeared less dense, greasy, and scruffy, resembling the K14-E7 phenotype more closely than that of K14-ΔN87βcat (Figure 2) [4]. Similar to K14-ΔN87βcat transgenic mice, they had grossly enlarged paws (Figure 2) and developed pilomatricomas, which are benign skin tumors [17]. Due to the combined effect of both transgenes, they exhibited a more severe skin phenotype than the K14-ΔN87βcat and K14-E7 single transgenics, especially around the snout and the eyelids (Figure 2). The K14-E7/ΔN87βcat-double transgenic animals had the lowest body weights (determined weekly) at the end of the study (Figure 3). This difference was highly significant when compared with those in other groups.

### Histopathology

**Figure 4:**
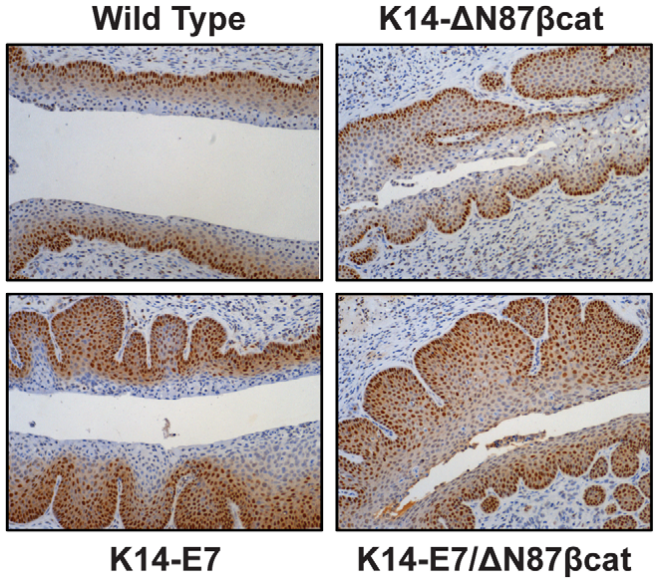
IHC Analysis of PCNA expression in cervical squamous epithelium. PCNA expression was restricted to the basal and innermost suprabasal layers in cervical sections of wild type and K14-ΔN87βcat transgenics, whereas in K14-E7 and K14-E7/ΔN87βcat transgenics, there was an incremental increase in expression within the multiple layers of the squamous epithelium. Cervical tissues were harvested at the endpoint of the study, after six months of estrogen treatment (0.05 mg/60 days, 7 months old). Images are at ×80 magnification.

Riley et al., observed cervical pathologies after 6 months of estrogen treatment (7 months of age) in K14-E7 transgenic animals [10]; therefore, we euthanized all animals at this same age in the four groups. Proximal vaginal, cervix, and both uterine horns were dissected en block and analyzed by histopathology. Proliferating cell nuclear antigen (PCNA) expression was detected by immunohistochemistry (IHC) to evaluate cell proliferation within the cervical squamous epithelium (Figure 4). PCNA expression was more abundant in K14-E7 and K14-E7/ΔN87βcat mice than wild type and K14-ΔN87βcat. In the latter two groups, PCNA expression was restricted to the basal and innermost suprabasal layers, whereas in K14-E7 and K14-E7/ΔN87βcat transgenics there was an incremental increase in the labeling index within the multiple layers of the squamous epithelium. However, PCNA expression in K14-E7/ΔN87βcat compared with K14-E7 mice was not significantly different.

**Figure 5:**
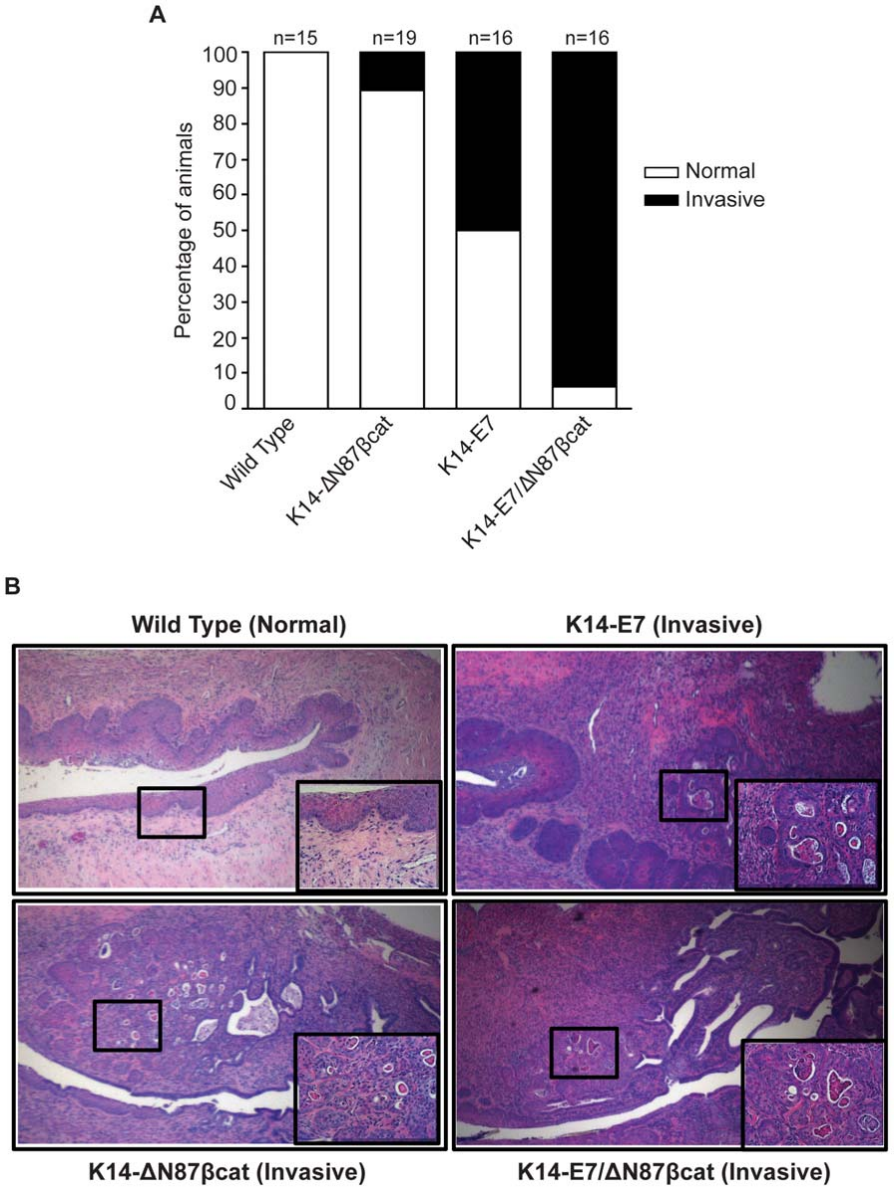
Histopathology. **A**) Distribution of cervical pathologies in the K14-E7/ΔN87βcat transgenics. Histopathological analysis of cervices from wild type, K14-ΔN87βcat, K14-E7, and K14-E7/ΔN87βcat groups is shown. **B**) Histopathological evaluation of mouse cervical tissues. A representative case from each genotype is shown. Cervical tissues were harvested at the endpoint of the study, after six months of estrogen treatment (0.05 mg/60 days, 7 months old), sectioned, and stained with H&E. Main panel and inset images are magnified by ×40 and ×160, respectively.

**Figure 6:**
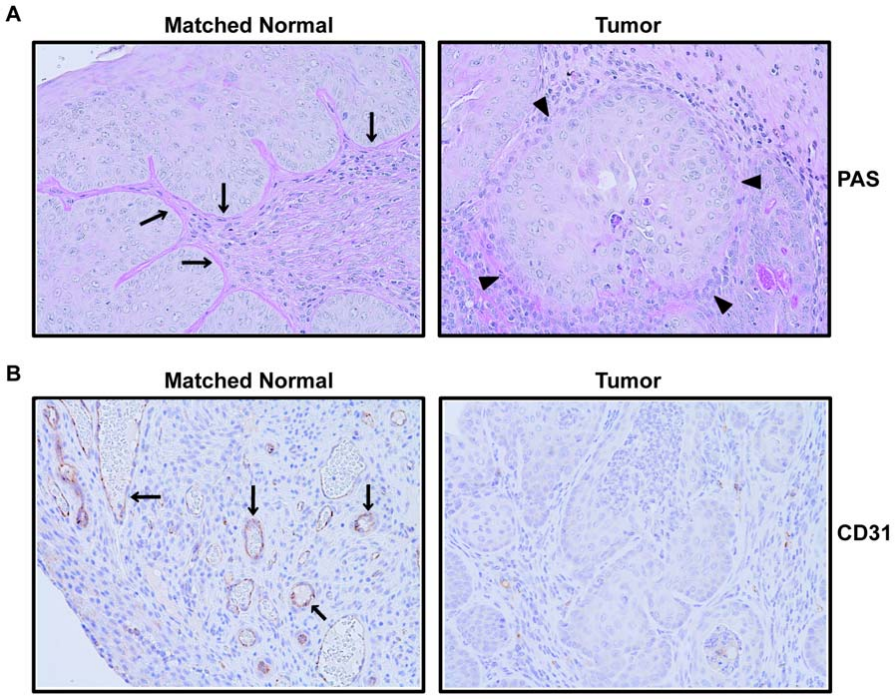
PAS staining and CD31 expression by K14-E7/ΔN87βcat transgenics. **A**) In normal cervical epithelium, PAS staining showed a continuous basement membrane (arrows), whereas tumors lacked this structure of the membrane (arrowhead). Both normal and tumor images are taken from the same section. **B**) Blood vessels were evaluated by CD31 (PECAM) expression using IHC. Vascular structures were present in normal tissues (arrows), but the invasive tumors did not contain any. Both normal and tumor images are taken from the same section. Cervical tissues were harvested at the study's end, after six months of estrogen treatment (0.05 mg/60 days, 7 months old). Images are at ×200 magnification.

Cervical pathologies were analyzed by hematoxylin and eosin (H&E) staining (Figure 5). Invasive cervical cancer developed in 11% (2/19) and 50% (8/16), respectively, in animals of the K14-ΔN87βcat and in the K14-E7 groups, but not in controls (0/15). Invasive cervical carcinomas developed in 94% (15/16) of the double transgenics, suggesting that the activation of Wnt pathway accelerates HPV16-E7-mediated cervical carcinogenesis (p = 0.02) (Figure 5A). A representative case from each genotype is given in Figure 5B. When estrogen pellets were omitted, we did not observe cervical cancer in either K14-E7 or K14-E7/ΔN87βcat (Figure S1). We performed Periodic Acid Schiff (PAS) staining to evaluate the basement membranes (Figure 6A) and analyzed CD31 (PECAM) expression by IHC to evaluate the blood vessels (Figure 6B) in all four groups. The most striking finding was that invasive tumors lacked a continuous basement membrane, nor did tumors contain any significant vascular structures.

### K14-HPV16/ΔN87βcat mice did not develop invasive cervical carcinomas

**Figure 7:**
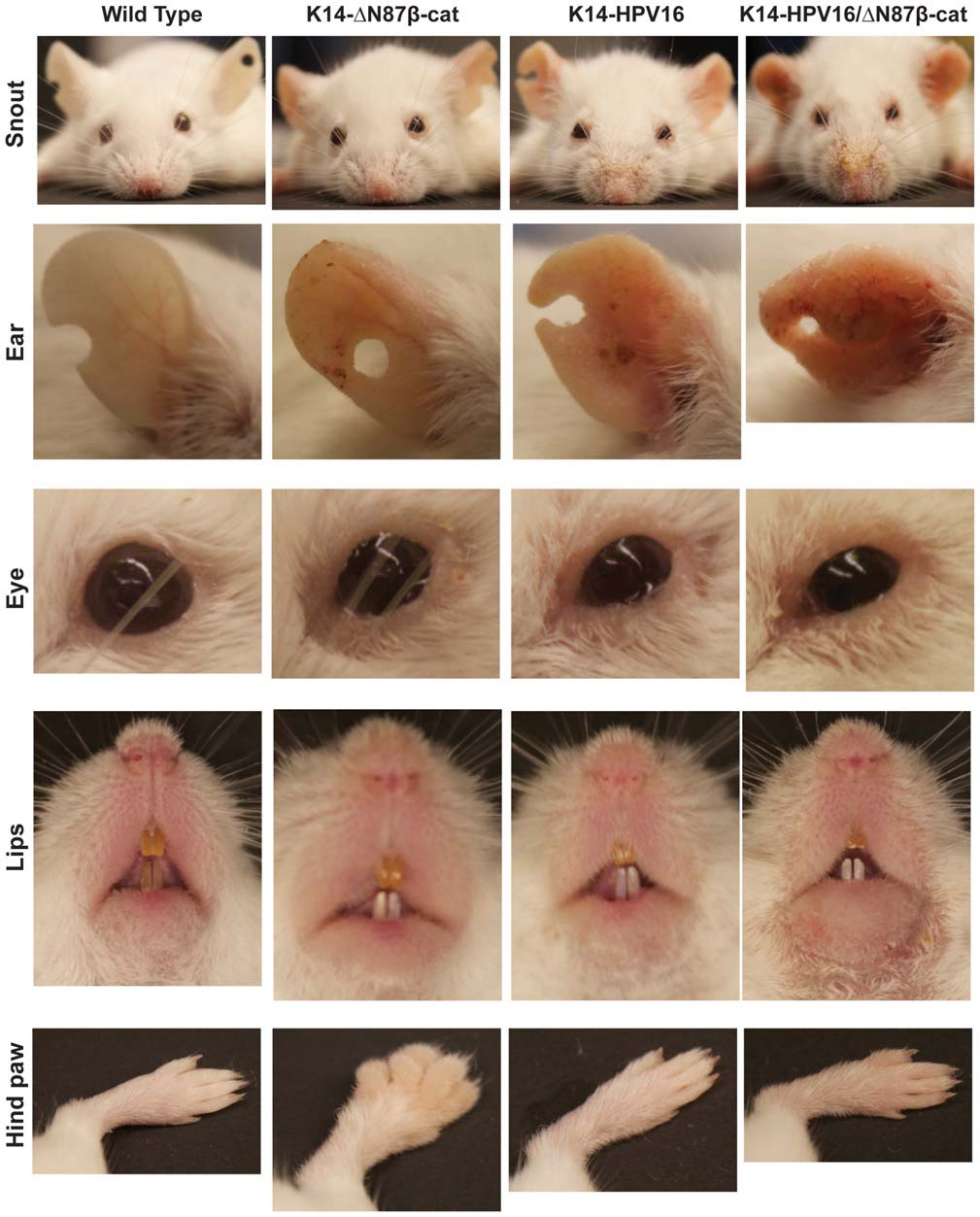
Phenotypic features of K14-HPV16/ΔN87βcat mice. K14-HPV16/ΔN87βcat double transgenic animals displayed certain phenotypic features characteristic of both K14-HPV16 and K14-ΔN87βcat transgenes. Photographs of snout, right ear, eye, lips and hind paw are given.

**Figure 8:**
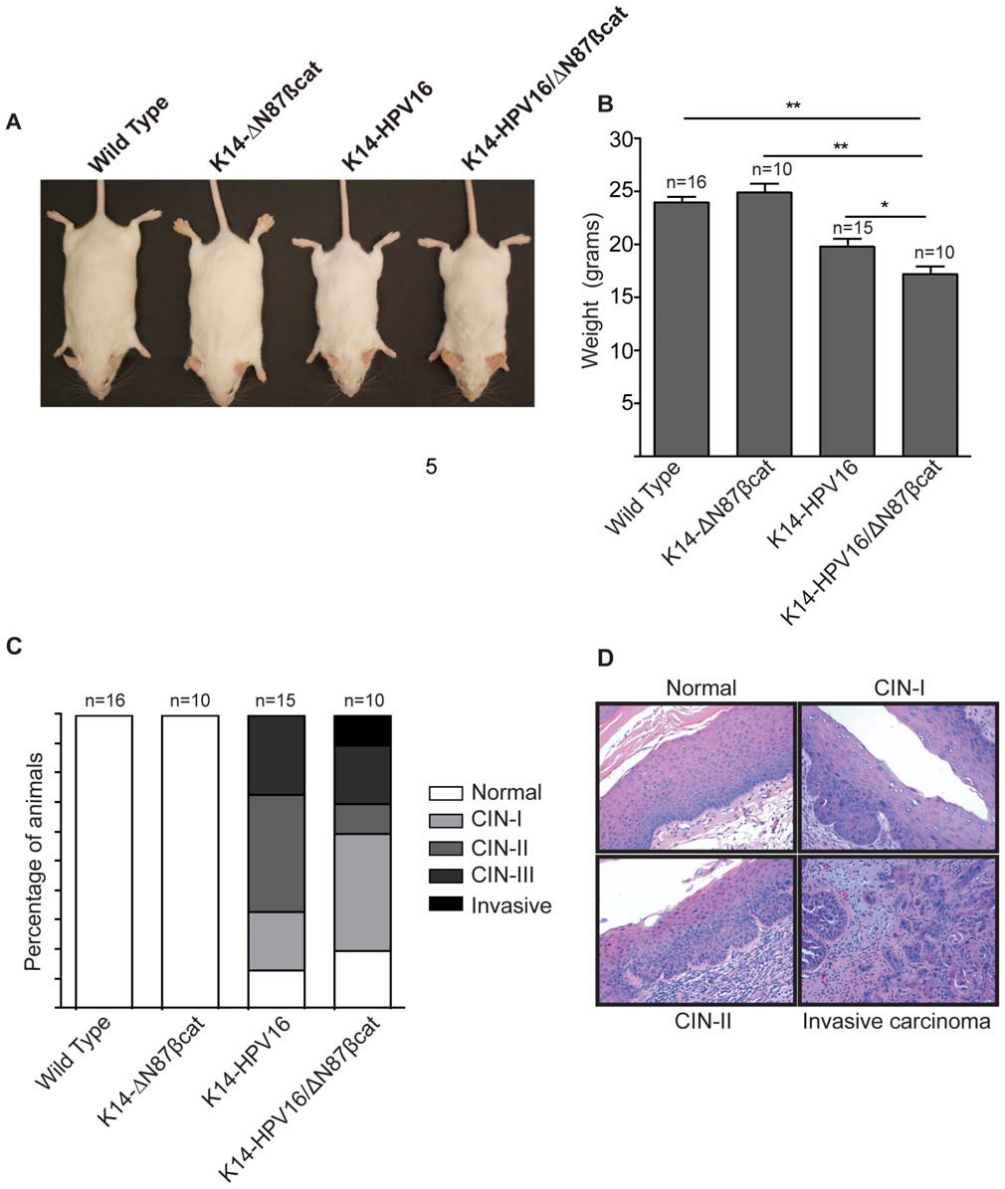
Body weights of K14-HPV16/ΔN87βcat mice. **A**) Whole body images of the four genotypes are given. **B**) Average body weights at approximately 5 months of age for the four experimental groups. (*p<0.05, **p<0.001) are shown. Bars represent mean values, and error bars represent standard deviations. Statistical analysis was done using the independent *t-test*. **C**) Distribution of cervical pathologies. Histopathological analyses of wild type, K14-ΔN87βcat, K14-HPV16, and K14-HPV16/ΔN87βcat cervical sections are shown. We did not detect cervical pathology in wild type (0/16) and K14-ΔN87βcat (0/10) animals. K14-HPV16 pathology varied as follows: cervical intraepithelial neoplasia-I (CIN-I), (3/15); CIN-II, (6/15); and CIN-III, (4/15). The frequency of normal cervical histology was (2/15). K14-HPV16/ΔN87βcat pathologies included CIN-I (4/10), CIN-II (1/10), CIN-III (2/10), and invasive tumor (1/10). Twenty percent exhibited normal histology (2/10). The average times of estrogen treatment of wild type, K14-ΔN87βcat, K14-HPV16, and K14-HPV16/ΔN87βcat mice were, respectively, 4.6, 4.6, 4.4, and 3.8 months. **D**) K14-HPV16/ΔN87βcat cervical histopathology. H&E stained slides of cervical tissues were analyzed for pathology. A representative case from each histopathological group is shown.

We also tested progression of cervical pathology in a second model employing K14-HPV16/ΔN87βcat transgenic mice. These animals were generated by crossing K14-HPV16 mice, which express E6 and E7 oncoproteins of the high risk HPV type 16 [5], [10] with K14-ΔN87βcat mice [17]. K14-HPV16/ΔN87βcat animals exhibited skin phenotypes comparable to those of K14-HPV16 and K14-ΔN87βcat animals (Figure 7) [5], [17]. Similar to the K14-E7/ΔN87βcat model, body weights of the K14-HPV16/ΔN87βcat double transgenic animals were lower than those of their littermates (Figure 8). In this group, K14-HPV16 mice showed increased cervical dysplasia compared to wild type (p<0.0001) and K14-ΔN87βcat mice (p = 0.001), but this phenotype was not augmented in K14-HPV16/ΔN87βcat animals (p = 0.59) (Figure 8C). We did not observe any invasive tumors in K14-HPV16 animals. A representative case from each histopathological group is shown in Figure 8D.

## Discussion

Several animal models have been established in attempts to understand the biology of HPV-mediated tumorigenesis, [3], [4], [5]. Two of the clinically relevant mouse models, K14-E7 and K14-HPV16 utilized the K-14 keratin promoter to target transgene expression to the stratified squamous epithelium, including the skin and the cervix. In these systems, both types of transgenic mice develop cervical pathologies following long-term estrogen treatment [8], [10]. We took advantage of these mice to test our hypothesis that activation of the Wnt pathway by β-catenin in the presence of HPV-E7 oncogene expression can accelerate the development of cervical cancer. We were able to generate transgenic mice (designated K14-E7/ΔN87βcat), which express the HPV16-E7 oncogene along with constitutively active β-catenin. We found that in this double-transgenic model, mice developed invasive cervical cancers with 94% penetrance as early as seven months of age. In striking contrast, these tumors occurred in only 50% of K14-E7 mice. The latter result conflicts with the studies of Herber *et al.*, which reported invasive cervical cancer in 80% of K14-E7 animals by 7 months of age [4]. This difference may be related to environmental or dietary factors.

The K14-ΔN87βcat mice used in our study were originally of the CD1 background and were backcrossed into FVB mice for 15 generations before they were used in our study. Therefore, we do not consider differences in genetic background to be a significant difference between our study and the one reported by Herber *et al*. Both CD1 and FVB strains are permissive to squamous epithelial malignancies [5]. Another factor related to this disparity might be diet, because phytoestrogens in the mouse diet can induce molecular and physiological changes in the uterus [18]. Phytoestrogens in laboratory diets can vary between formulations and from batch to batch of the same formulation [19]. Phytoestrogens can also mimic the properties of mammalian estrogens. Since estrogen dose is an important parameter in the development of cervical cancer [8], dietary phytoestrogens can influence estrogen-regulated processes and strongly influence studies on comparative carcinogenicity. The animals in our study were fed the PMI Irradiated Rodent 5053 diet from Harlan Laboratories, which contains 380 ppm of phytoestrogens. Although our present results differ from other reports regarding tumor penetrance [4], all of our mice possessed the same genetic background (all F1 littermates), diet, and housing environment. Therefore, our study was internally controlled. We feel confident, therefore, in concluding that our results support our main hypothesis that β-catenin can accelerate cervical cancer progression.

We observed an intriguing difference in total body weights between mice in the groups (Figure 3). However, we were unable to determine the underlying mechanism. Because K14-E7/ΔN87βcat mice had the lowest body weights and the highest tumor incidence, tumor-related cachexia might have been involved. However, when we compared the weights of K14-E7 animals with tumors (n = 8) to ones without (n = 8), we did not detect a statistically significant difference (Figure S2). We could not perform the same comparison for the other groups because the number of animals was insufficient for meaningful statistical analysis. We believe that it is reasonable to conclude that the weight difference resulted from the progressively worsening combined skin phenotype. We initially supposed that the double transgenics would have difficulty in eating or drinking. However, when we assessed food and water intake by members of each group for 6 weeks, we did not observe any statistically significant differences (Figure S3). Therefore, only the combined skin phenotype remains as a plausible explanation.

Two out of 19 K14-E7/ΔN87βcat, but none of the K14-HPV16/ΔN87β mice (0/10) developed invasive tumors. The K14-ΔN87βcat mice that constitute both double transgenic models are not derived from same genetic background and therefore cannot be compared with each other. Since only F1 littermates were used in the study, K14-ΔN87βcat littermates of K14-HPV16/ΔN87βcat were derived from one of each parental heterozygote, namely K14-ΔN87βcat and K14-HPV16. In contrast, K14-ΔN87βcat littermates of the K14-E7/ΔN87βcat were derived from the parental heterozygotes K14-ΔN87βcat and K14-E7. However, since both K14-HPV16 and K14-E7 possess the FVB/N background, one would have expected no phenotypic difference. To evaluate the genetic backgrounds of K14-HPV16 and K14-E7 mice, we performed SNP analyses on three animals from each group. A 1449 loci medium density SNP linkage analysis showed that K14-E7 and K14-HPV16 mice, respectively, were 100% and 99.83% FVB/NTac. These findings suggested that the alteration in the genetic background of K14-HPV16 animals might have been responsible for the differences between the two K14-ΔN87βcat groups. Furthermore, the same genetic alteration might account for why we observed only dysplasias in K14-HPV16/ΔN87βcat mice compared with invasive cancer and dysplasias no in K14-E7/ΔN87βcat mice.

We performed IHC to evaluate nuclear β-catenin levels (Figure S4). We did not observe significant nuclear β-catenin staining signal intensity in sections prepared from either group group (Figure S4). The endogenous level of β-catenin is very high in the mouse cervix (Figure 1B), but IHC was not sensitive enough to detect protein expressed from transgene. However, our published data and other IHC studies demonstrate that β-catenin localizes to the cytoplasm or nucleus in more than two thirds of human invasive cervical carcinoma samples [14], [15], [16], suggesting an active canonical Wnt signaling pathway.

We believe that two mechanisms might account for observed Wnt pathway activation in cervical cancer. Mutations in the β-catenin gene are common in colon cancer, whereas β-catenin mutations in cervical cancer are rare, which suggests activation of the Wnt pathway upstream of β-catenin in cervical carcinoma [20]. Epigenetic changes in Wnt pathway regulators in cervical tumor samples and cell lines, as well as in other cancers have been reported, which can explain the activation of the canonical Wnt pathway in the absence of β-catenin mutations [21], [22], [23], [24], [25], [26], [27]. Alternatively, HPV oncogenes might directly modulate the Wnt pathway. One recent report provides evidence that HPV16 E6 is able to augment β-catenin/TCF-dependent transcription induced by Wnt3a or β-catenin expression. Because this augmentation is not associated with GSK-3 β activity or major alterations in the levels, stability, or cellular distribution of β-catenin, the authors suggest that this relates to activation of ubiquitin ligase E6AP by E6 [28]. In another study, Rampias *et al.* (2010) reported that in oropharyngeal cancer cells, HPV16 E6 and E7 oncogenes are involved in nuclear accumulation of β-catenin and activation of Wnt signaling mediated by the ubiquitin/proteosome pathway [29].

Our study provides a potential link between activation of the Wnt signaling pathway and its contribution to HPV-mediated cervical cancer. These results indicate that activation of the canonical Wnt pathway might represent secondary events that are required for malignant transformation of HPV-infected epithelial cells. Targeting the canonical Wnt pathway may therefore provide the basis for developing clinical interventions to prevent disease progression in populations at risk for HPV infection and to treat advanced cervical cancers.

## Materials and Methods

### Materials

Unless stated otherwise, all chemical reagents were purchased from Sigma (St. Louis, MO).

### Ethics Statement

Georgetown University's Institutional Animal Care and Use Committee (GUACUC) approved all animal studies, protocol IDs 07-064 and 10-029.

### Transgenic Mice

The generation of K14-E7, K14-HPV16 and K14-ΔN87βcat transgenic mice has been described [4], [5], [17]. All three transgenic mouse models were maintained as heterozygotes. K14-E7 and K14-HPV16 (FVB) mice were acquired from the Mouse Models of Human Cancers Consortium (MMHCC) of the National Cancer Institute. K14-ΔN87βcat mice were kindly provided by Dr. E. Fuchs, Rockefeller University. K14-ΔN87βcat transgenic mice (CD1 background) were first backcrossed with FVB mice for 15 generations. Double transgenic mice were generated by crossing K14-ΔN87βcat mice with either K14-E7 or K14-HPV16 mice. Only F1 generation female animals were used.

### Genotyping and genetic characterization

Offspring were screened for the presence of transgenes by PCR amplification of genomic DNA isolated from mouse-tails at weaning. Primers specific for the K14-E7 (F:5′-GGCGGATCCTTTTTATGCACCAAAGA-3′, R:5′-CCCGGATCCTACCTGCAGGATCAGC-3′), K14-HPV16 (F:5′-AGAACTGCAATGTTTCAGGACCCACAG-3′, R:5′-TCTGCAACAA GACATACATCGACCGG-3′) and K14-ΔN87βcat (F:5′-TCCCACTAATGTCCAGCG-3′, R:5′-CGCGGCATGCAGGTACC-3′) transgenes were used for genotyping. PCR reactions were initiated by denaturation at 94°C for 7 min, followed by 1 min at 94°C, 1 min at 55°C, and 1 min at 72°C, for 35 cycles. The reaction was completed with a final extension step at 72°C for 10 min. Amplifications with *p53*-specific primers were used as a positive control. Samples were run through a 2% agarose gel and then imaged.

Genetic characterization of the K14-E7 and K14-HPV6 mice were performed using 1449 dense Single Nucleotide Polymorphism (SNP) Marker Panel (GENCON Panel 4, Taconic, Rensselaer, NY, USA).

### ΔN87βcat Protein Expression

Heart, skin, and cervical tissues were snap frozen in liquid nitrogen, ground to a powder, lysed with a buffer consisting of 20 mM Tris-HCl, pH 7.5, 150 mM NaCl, 2.5 mM EDTA, 10 mM NaF, 10 mM Na_2_P_2_O_7_, 1 mM Na_3_VO_4_, 10 µM aprotinin, 20 µM leupeptin, and 1 mM PMSF. Total lysates (40 µg for heart and cervix and 2 µg for skin samples) were resolved on SDS-PAGE gel. Tissue-specific expression of human ΔN87βcat was determined by immunoblot analysis with 250 ng/ml of an anti-β-catenin antibody (BD Transduction Laboratories™, San Jose, CA) as described previously [30]. Heart tissue was used as negative control because K14 promoter activity was not detected in this tissue.

### Hormone Treatment

One-month-old virgin female mice were anesthetized using 5% isofluorane, and pellets of 17 β-estradiol (Innovative Research of America, Sarasota, FL) delivering estrogen at a dose of 0.05 mg/60 days were placed subcutaneously in the dorsal skin, near the shoulder fat pads. Animals received a new pellet every 60 days.

### Histopathological evaluation of mouse cervical tissues

Each organ was sectioned through its entire depth to generate 5 µM thick slices. A representative of 20 sections was stained with H&E. Histopathology was performed by pathologists B.K. (K14-E7/ΔN87βcat mice) and C.P.C. (for K14-HPV16/ΔN87βcat mice). Samples were evaluated by a single blind method.

### Immunohistochemistry

Five-micron sections from formalin-fixed paraffin embedded cervical tissues were deparaffinized with xylenes and sequentially rehydrated using a graded alcohol series. Heat-induced epitope retrieval was performed by immersing the tissue sections at 98°C for 20 min in 10 mM citrate buffer (pH 6.0), 0.05% Tween-20. IHC was performed using the VectaStain Kit from Vector Labs (Burlingame, CA) according to manufacturer's instructions. Briefly, slides were treated with 3% hydrogen peroxide and 10% normal serum, and incubated with a PCNA antibody (1∶600 dilution, Santa Cruz Biotechnology, Inc., Santa Cruz, CA) for 1 h at room temperature (RT). Slides were treated with the appropriate species-specific biotin-conjugated secondary antibody (Vector Labs), Vectastain ABC reagent (Vector Labs), DAB chromagen (Dako, Carpinteria, CA), and then counterstained with hematoxylin (Harris Modified Hematoxylin, Fisher Scientific, Pittsburgh, PA) (1∶17 dilution) for 2 min at RT, blued in 1% ammonium hydroxide for 1 min at RT, dehydrated as described above, and mounted with Acrymount (StatLab Medical Products, McKinney, TX). Sections incubated with the secondary antibody only were used as negative controls. β-catenin (Cell Signaling, Danvers, MA, USA) and CD31 (PECAM, Santa Cruz) IHCs were done in a similar manner with both antibody dilutions as 1∶200. All photomicrographs shown here and in all other figures were taken using a Nikon E600 microscope.

### Periodic Acid-Schiff (PAS) Staining

Periodic Acid-Schiff reaction (PAS) was performed as described by Bancroft and Gamble with minor modifications [31]. Briefly, five micron sections from formalin fixed paraffin embedded tissues were de-paraffinized with xylenes and rehydrated through a graded alcohol series. Sections were treated with 0.5% periodic acid, microwaved in Schiff's reagent (Sigma) for 40 seconds, counterstained with Mayer's hematoxylin and blued in ammonium hydroxide. Sections were dehydrated, cleared in xylenes and mounted with Acrymount.

### Statistical Analysis

Statistical analyses were performed using Prism version 4.0 (GraphPad Software, La Jolla, CA). Animal weights between groups were compared using an independent *t*-test. The Fisher Exact test was used to compare rates of invasive cervical cancer and the Wilcoxon rank-sum test was used to compare the pathology data between experimental groups. Statistical significance was defined as p<0.05.

## Supporting Information

Figure S1 **Cervical phenotypes in K14-E7/ΔN87βcat transgenics that were not treated with estrogen.** **A**) Cervical tissues were harvested at the study's end when the animals were 7 months old. Histopathological analysis of cervical sections from wild type, K14-ΔN87βcat, K14-E7 and K14-E7/ΔN87βcat mice are shown. **B**) Histopathological evaluation of mouse cervical tissues. H&E-stained slides from cervices of mice not treated with estrogen. A representative case from each genotype is given. Images are at ×200 magnification.
(TIF)

Figure S2 **Difference in total weight of animals was not related to the presence of tumors.** Average body weights of K14-E7 mice with and without tumors were compared. The difference in between these two groups was non-significant. Statistical analysis was evaluated using independent *t*-test.
(TIF)

Figure S3 **Dietary intake.** Data for food and water are shown in panels (A) and (B), respectively. Bars represent mean values, and error bars represent standard deviations.
(TIF)

Figure S4 **Evaluation of nuclear βcatenin protein levels using IHC in K14-E7/ΔN87βcat model.** We did not observe significant nuclear β-catenin signal intensity in sections prepared from mice cervices. Cervical tissues were harvested at the study's end, after six months of estrogen treatment (0.05 mg/60 days, 7 months old). Left column is at ×100 and right column is at ×200 magnification.
(TIF)
