# Supplementary figures and images for: Beta-Catenin Accelerates Human Papilloma Virus Type-16 Mediated Cervical Carcinogenesis in Transgenic Mice
Source: PLoS One. 2011 Nov 7;6(11):e27243. doi: 10.1371/journal.pone.0027243 (PMC3210148; doi:10.1371/journal.pone.0027243)

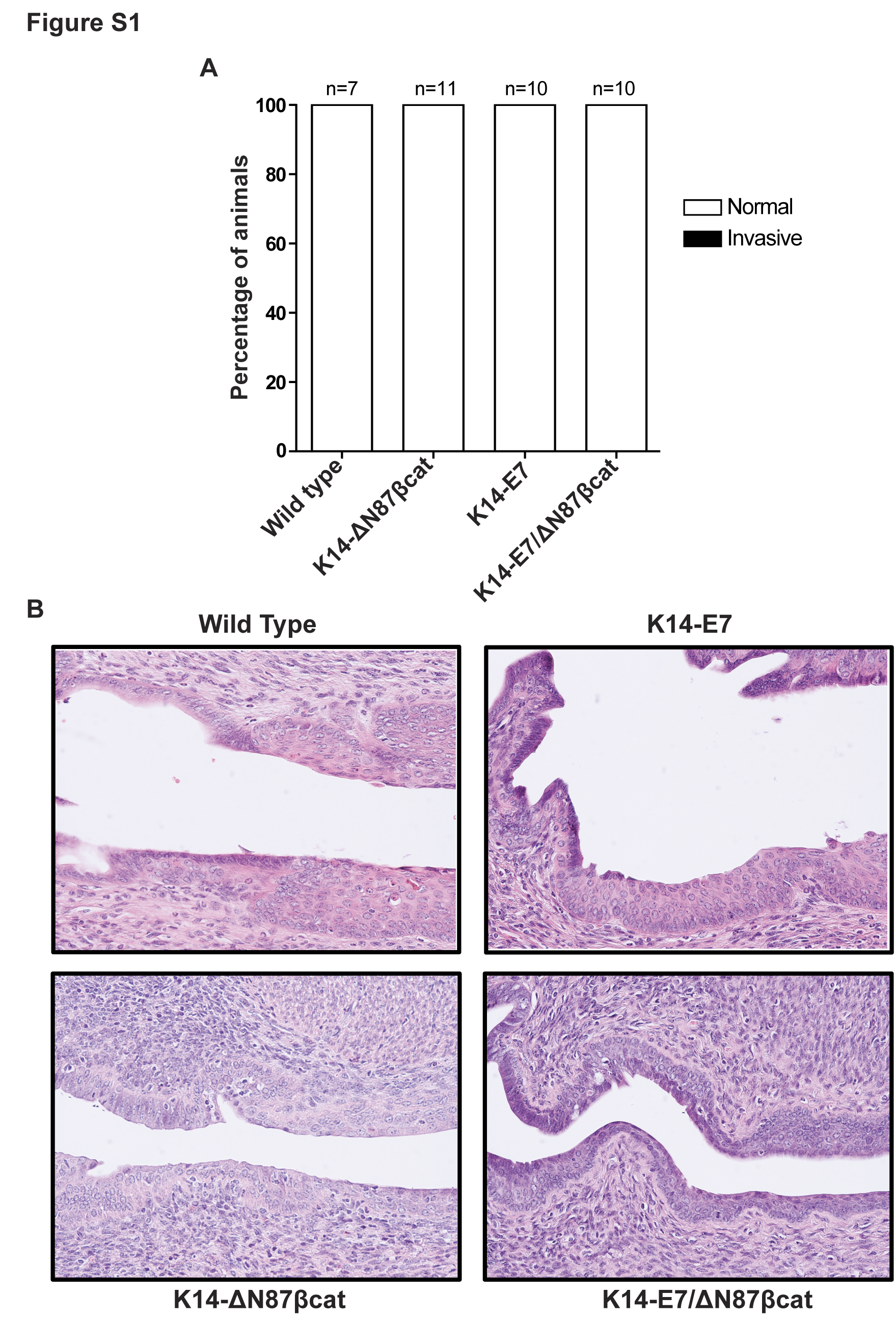

Supplement: Figure S1 — Cervical phenotypes in K14-E7/ΔN87βcat transgenics that were not treated with estrogen. A) Cervical tissues were harvested at the study's end when the animals were 7 months old. Histopathological analysis of cervical sections from wild type, K14-ΔN87βcat, K14-E7 and K14-E7/ΔN87βcat mice are shown. B) Histopathological evaluation of mouse cervical tissues. H&E-stained slides from cervices of mice not treated with estrogen. A representative case from each genotype is given. Images are at ×200 magnification. (TIF) [file pone.0027243.s001.tif]

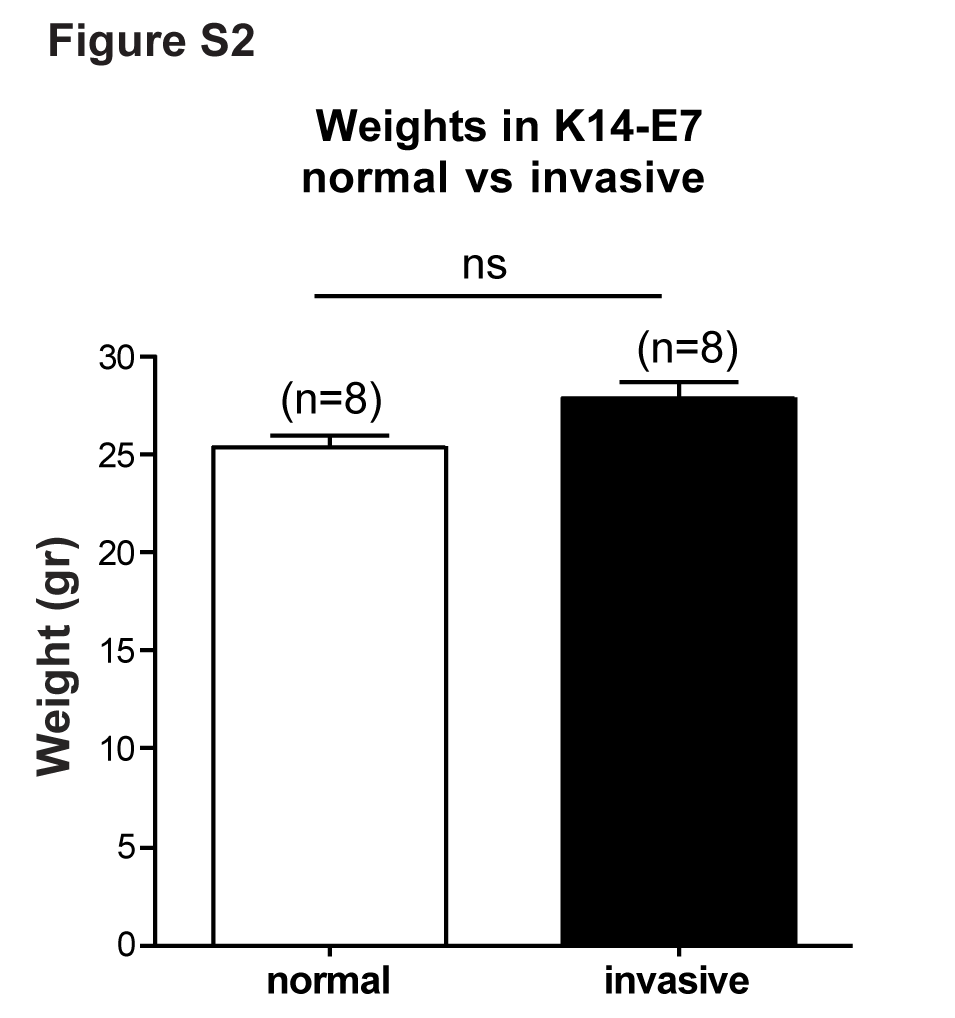

Supplement: Figure S2 — Difference in total weight of animals was not related to the presence of tumors. Average body weights of K14-E7 mice with and without tumors were compared. The difference in between these two groups was non-significant. Statistical analysis was evaluated using independent t-test. (TIF) [file pone.0027243.s002.tif]

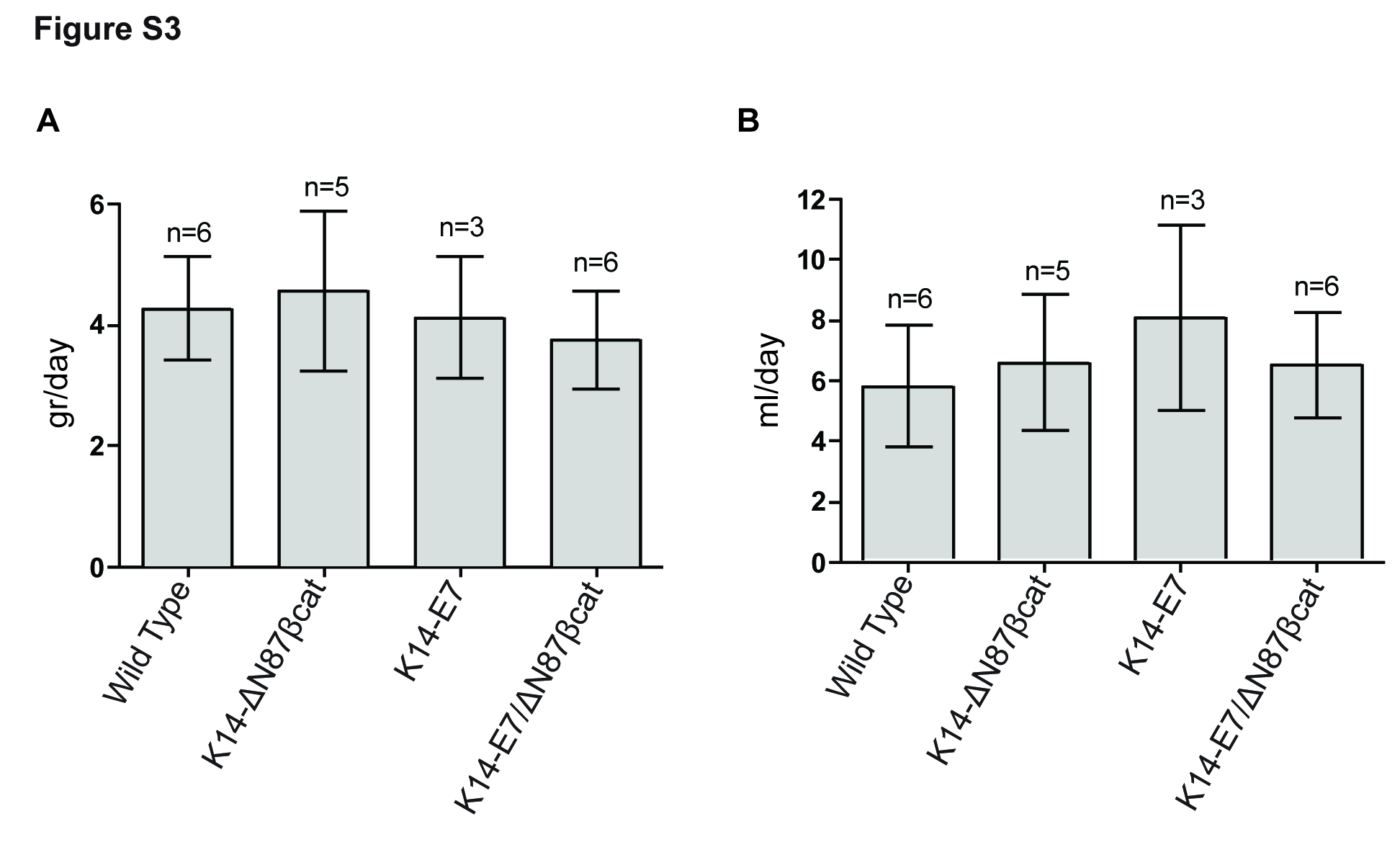

Supplement: Figure S3 — Dietary intake. Data for food and water are shown in panels (A) and (B), respectively. Bars represent mean values, and error bars represent standard deviations. (TIF) [file pone.0027243.s003.tif]

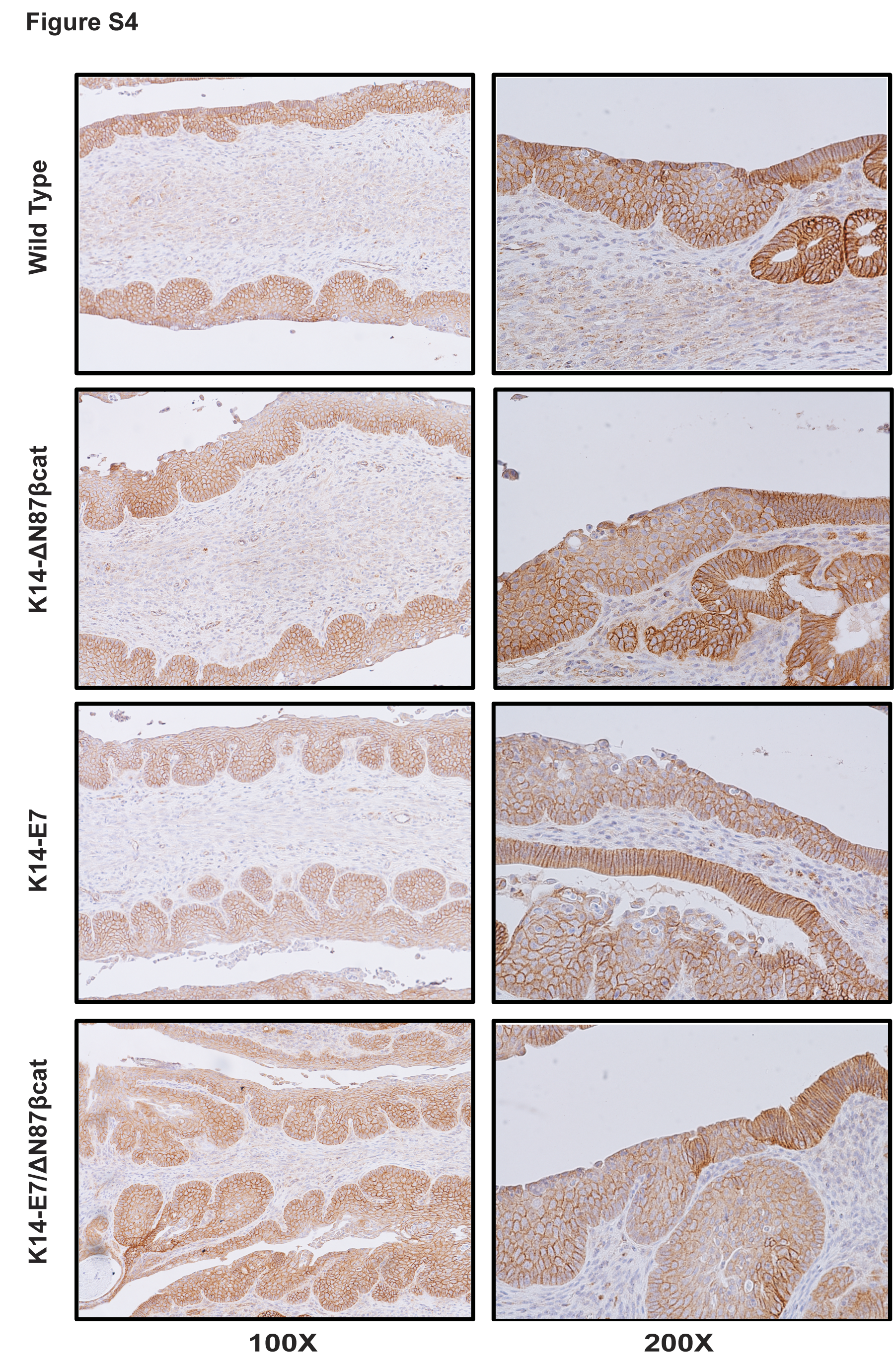

Supplement: Figure S4 — Evaluation of nuclear βcatenin protein levels using IHC in K14-E7/ΔN87βcat model. We did not observe significant nuclear β-catenin signal intensity in sections prepared from mice cervices. Cervical tissues were harvested at the study's end, after six months of estrogen treatment (0.05 mg/60 days, 7 months old). Left column is at ×100 and right column is at ×200 magnification. (TIF) [file pone.0027243.s004.tif]
